# Moderating Effect of Nurse’s Character on the Relationship between Attitudes toward Nursing Care of the Dying and Performance of Terminal Care in South Korea

**DOI:** 10.3390/healthcare9091195

**Published:** 2021-09-10

**Authors:** Kawoun Seo

**Affiliations:** Department of Nursing, Joongbu University, Goyang 32713, Korea; kwseo@joongbu.ac.kr

**Keywords:** hospices, terminal care, character, attitude toward death, nurses

## Abstract

This study aimed to investigate the moderating effect of nurses’ characteristics on the relationship between attitudes toward nursing care and terminal care performance of hospice nurses. The participants included nurses working in hospice care units in general hospitals in South Korea. Data collected from August 1 to 31, 2020 were analyzed using *t*-test, ANOVA, Pearson′s correlation coefficients, and hierarchical multiple regression. The performance of terminal care was positively correlated with attitudes toward nursing care of the dying (r = 0.45, *p* < 0.001) and nurses’ characteristics (r = 0.60, *p* < 0.001). There was also a positive correlation (r = 0.58, *p* < 0.001) between attitudes toward nursing care for dying patients and nurses′ characteristics. Nurses’ characteristics had a significant moderating effect on the relationship between attitudes toward nursing care of the dying and performance of terminal care. This means that the nurses’ character had a buffering effect on the relationship between attitudes toward nursing care of the dying and performance of terminal care in hospice care units. These findings suggest that continuous and repetitive educational programs on terminal care need to be enhanced, and strategies to strengthen attitudes toward nursing care of the dying and nurses’ character need to be included in these programs.

## 1. Introduction

Although hospice palliative care commenced in Korea in 1965, progress has been slow owing to the failure to establish a stable financial foundation while conducting hospice palliative care from a religious and private perspective [1]. However, interest in hospice palliative care has been increasing with the growing awareness of the importance of end-of-life care in the context of a growing, aging population and the implementation of the national health insurance hospice benefit system [1,2]. In addition, with the establishment of the Act on the Determination of Lifetime Medical Care for Patients in the Hospice, Palliative Care, and on the Dying Process, the user rate is expected to increase as the service scope expands to include terminal cancer as well as acquired immunodeficiency syndrome, chronic obstructive pulmonary disease, and chronic cirrhosis [1,3].

Hospice palliative care involves the creation of a multidisciplinary team to improve the quality of life of terminally ill patients, along with that of their families. The aim is to provide holistic care, including physical, emotional, spiritual, legal, and financial counseling [4]. Most nurses in hospice palliative care units are responsible for providing nursing care to terminally ill patients. They provide physical care to reduce pain and discomfort caused by the symptoms, as well as psychological or spiritual counseling for those suffering from fear of death. Spiritual nursing also helps patients to improve their quality of life and maintain their dignity [5]. Therefore, for terminally ill patients, end-of-life nursing provided by hospice nurses plays a very important role in improving their quality of life. Among the factors that influence the performance of terminal care by hospice nurses, the most significant is the attitude toward nursing care for dying patients.

Attitudes toward nursing care for dying patients refer to the positive or negative emotions and perceptions of nurses who provide nursing care to these patients [6]. Patients and their families who are assigned nurses with positive attitudes toward nursing care are considered to have a high quality of life and are likely to face death more gracefully [7]. Furthermore, attitudes toward nursing care for dying patients were found to significantly influence the performance of terminal care by intensive care unit nurses [8], and it is also an important influencing factor in the performance of terminal care of cancer ward nurses [9]. However, some of these nurses encounter difficulties in performing terminal care due to the lack of knowledge, experience, or values, a limited understanding of end-of-life patients, or communication problems with other medical personnel [10]. In addition, nurses said that they found terminal care challenging as they experienced spiritual conflicts caused by anxiety or skepticism about death [3]. In particular, the dearth of nurses, workload processes, and lack of communication hinders the performance of terminal care [8]. However, to maintain the quality of life and dignity of individuals who use the hospice ward, it is critical to launch efforts to improve the performance of hospice nurses’ terminal care.

Meanwhile, clinical nursing character refers to desirable attitudes and behaviors that active clinical nurses should possess [11], including having a personality that is suitable for caring for a patient. These include a sense of responsibility, a passion for improving work ability, and maintaining composure in situations where emotions are high. Other qualities include coping, empathy, the ability to work smoothly in cooperation with colleagues, behaviors that respect the personality of others, and recognition of the existence or value of others [11]. These qualities enable nurses to overcome barriers to terminal care. Effectively, nurses’ competence in terms of their end-of-life nursing attitude is expressed through empathy [12], as well as their ability to maintain their composure despite the stress arising from terminal care or to change one’s perception of death through their experiences to realize the meaning of care for end-of-life patients [13]. Considering the above, it can be predicted that a nurse’s character, particularly having a positive attitude toward nursing care of the dying, can serve as a buffer and help in overcoming obstacles to performing effective terminal care. However, there are no comprehensive studies on the relationship between attitudes toward nursing care of the dying and performance of terminal care among hospice nurses in Korea, while studies on nurses’ characteristics are limited.

Therefore, this study aimed to examine whether a nurse’s character has a moderating effect on the relationship between attitudes toward nursing care of the dying and the performance of hospice nurses’ terminal care. The results of this study can be used as basis data for developing an intervention program to enhance the performance of hospice nurses’ terminal care in Korea.

## 2. Materials and Methods

### 2.1. Design 

This study explores the moderating effect of a nurse’s character on the relationship between attitudes toward nursing care of the dying and terminal care performance of hospice nurses. 

### 2.2. Participants and Data Collection

The participants of this study included 158 hospice nurses who worked in hospice wards of general hospitals located in D city and C and K provinces for more than three months. They understood the purpose of the study and voluntarily agreed to participate.

The data for this study were collected using a self-report questionnaire consisting of topics such as general characteristics, clinical career, attitudes toward nursing care of the dying, performance of terminal care, and the character of nurses. After explaining the purpose of the study and the contents of the survey, the questionnaires were distributed to hospice nurses in the nursing department of each hospital. The data collection period was from 1 to 31 August 2020.

### 2.3. Measurements

The questionnaire consists of eight questions regarding the general characteristics and career, 20 items that evaluate attitudes toward nursing care of the dying, 21 items that measure the performance of terminal care, and 53 items that evaluate the nurse’s character, for a total of 102 items.

#### 2.3.1. Attitudes toward Nursing Care of the Dying (ANCD)

To evaluate attitudes toward nursing care of the dying, the Frommel Attitudes Toward Nursing Care of the Dying Scale (FATCOD) developed by Frommelt and translated by Cho and Kim was used [14,15]. The original tool consisted of 20 questions regarding nurses’ attitudes toward patients and 10 questions on family care for terminally ill patients. However, only 20 questions on nurses’ attitudes were used in this study. Each question was answered on a 4-point Likert scale. At the time of tool development [14], the reliability of the tool was Cronbach’s α = 0.98, 0.94 in the study by Cho and Kim [15], and in this study, the reliability of the tool was Cronbach’s α = 0.79.

#### 2.3.2. Performance of Terminal Care

The performance of terminal care was measured using the performance of the terminal care tool developed by Park and Choi and revised by Chung [16,17]. This tool has 21 questions overall: eight questions each for the physical and psychological domains, and five questions regarding spiritual aspects. Responses were answered using a 4-point Likert scale. At the time of tool development [16], the reliability of the tool was Cronbach’s α = 0.96, 0.80 in the study by Chung [17], and, in this study, the reliability of the tool was Cronbach’s α = 0.89.

#### 2.3.3. Nurse’s Character (ND)

The nurses’ character was measured using a nurse’s character tool developed by Park [11]. This tool consists of 53 questions. Each question was answered using a 5-point Likert scale, and the higher the total score, the better the personality of the nurse. In Park’s study [11], the reliability was Cronbach’s α = 0.94; in this study, the reliability of the tool was Cronbach’s α = 0.95.

### 2.4. Data Analysis

The collected data were analyzed using the SPSS/WIN 24.0. Descriptive statistics were used for general characteristics of the study participants, and Pearson’s correlation coefficients were used to determine the correlation between ANCD, performance of terminal care, and ND. The moderating effect of NC on the relationship between ANCD and performance of terminal care of hospice nurses was analyzed through hierarchical multiple regression. Reliability was measured using Cronbach’s α.

## 3. Results

### 3.1. Participants’ Characteristics

The general characteristics of the study participants are listed in Table 1. The average age of the participants in this study was 39.4 years (±10.8) with those in their 30s or younger accounting for 30.4% of all participants. Among the participants, 47.5% had attained a bachelor’s degree, while 39.9% had attained a master’s degree. Among the participants, 68.4% engaged in religious activities, 48.1% were married, and 22.2% lived alone. The average hospice nurse’s career was 3.98 (±4.16) years, whereas the majority of the nurses (33.5%) had less than one year of experience. Only 24.1% of the nurses were hospice specialist nurses, and 66.5% said they were satisfied with their jobs.

The mean score of attitudes toward nursing care of the dying in this study was 3.22 (±0.31) points. Meanwhile, the mean score for the performance of terminal care was 3.18 (±0.33) points. The mean score for nurses’ character was 4.06 (±0.40) points.

### 3.2. Differences in the Performance of Terminal Care According to General Characteristics 

There were significant differences in the performance of terminal care based on the general characteristics of the participants according to age (F = 7.04, *p* < 0.001), religion (t = 4.26, *p* < 0.001), marital status (t = 7.04, *p* < 0.001), career of hospice nurses (F = 4.95, *p* = 0.003), hospice specialist nurses (t = 2.06, *p* = 0.004), and job satisfaction (F = 5.09, *p* = 0.007) (Table 2).

### 3.3. Correlation between Independent Variables

ANCD had a positive correlation with the performance of terminal care (r = 0.45, *p* < 0.001) and NC (r = 0.58, *p* < 0.001). There was also a positive correlation between the performance of terminal care and NC (r = 0.60, *p* < 0.001).

### 3.4. Moderating Effects of Nurse’s Character in Relation to Attitude toward Nursing Care of the Dying and Performance of Terminal Care

A multi-collinearity test was conducted before hierarchical regression analysis to confirm the moderating effects of NC on the relationship between ANCD and the performance of terminal care. The Durbin–Watson value was 2.019, and there was no autocorrelation of error. The limit of tolerance was 0.58–0.95, all above 0.1, and the variance expansion coefficients of all variables ranged from 1.06–1.73, not exceeding 10. Therefore, there was no multicollinearity among the variables.

The explanatory power of Model 1, in which only control variables were inputted with performance of terminal care as a dependent variable, was 14.8%, and none of the variables had a significant influence (F = 4.88, *p* < 0.001). In Model 2, ANCD was added as an independent variable to the control variable of Model 1 and to understand its effect. The explanatory power increased to 24.3%, and ANCD (β = 0.35, *p* < 0.001) had a significant effect on the performance of terminal care (F = 7.25, *p* < 0.001). In Model 3, NC was added to the independent variable in Model 2 to determine its moderating effect. The explanatory power of Model 3 was 37.2%, and only NC (β = 0.49, *p* < 0.001) had a statistically significant effect (F = 11.25, *p* < 0.001). Finally, in order to examine the moderating effect, the interaction variable between ANCD and NC was added to the independent variable of Model 3. The explanatory power of Model 4 increased to 41.3%, and among the independent variables, religion (β = 0.15, *p* = 0.037), ANCD (β = 0.18, *p* = 0.032), NC (β = 0.51, *p* < 0.001), and the interaction between ANCD and NC (β = 0.22, *p* < 0.001) were found to be statistically significant variables (F = 11.99, *p* < 0.001). As a result of testing the moderating effect of NC on the relationship between ANCD’s effect on performance of terminal care, it was found that explanatory power gradually increased to 24.3% in Model 2, 37.2% in Model 3, and 41.3% in Model 4. Since the *p*-value is also less than 0.05 in all models, the moderating effect can be confirmed. In conclusion, NC was found to have a moderating effect between the ANCD and performance of terminal care. In other words, with regard to the relationship between ANCD and performance of terminal care, it was found that the higher the NC, the greater the effect of the ANCD on the performance of terminal care. Therefore, the NC greatly amplified the attitude toward nursing care for dying patients (Table 3).

## 4. Discussion

The terminal care performance of hospice nurses is critical in enhancing the quality of life of hospice patients, alleviating their physical and psychological distress, and ensuring a peaceful death. Therefore, this study was conducted to confirm the moderating effect of NC, an individual competency, on the relationship between ANCD and the performance of terminal care by hospice nurses.

The mean score of hospice nurses for ANCD was 3.22 points (range 1–4 points), which is higher than that reported by Frommelt, who stated an average of 2 points at the time of tool development [14]. In addition, this score was higher than the 2.83 points found in a study of intensive care unit nurses [8] and the 2.86 points found in a study of general hospital nurses [12]. A possible reason is that a relatively higher proportion of patients require terminal care in hospice wards than in general wards. In addition, the goal of the hospice palliative unit is to improve the quality of life of patients while aiding them in achieving a peaceful death as compared to the intensive care unit, which focuses more on the recovery and resuscitation of the patient. However, in a previous study targeting hospice palliative ward nurses [18], the level of ANCD was similar to that of a study targeting intensive care unit nurses. In this study, unlike previous studies [18], there is a possibility that the score was relatively high because the nurses’ attitudes toward the patient’s family were excluded from among the research tools. Since most nurses provide patient-centered care, there is a possibility that the proportion given to the patient’s family is relatively lower than that to the patient. However, considering that the patient’s family also faces various difficulties [19], further research on ANCD, including the patient’s family, should be conducted in subsequent studies.

The mean score for NC was 4.06 points (range 1–5 points), which was higher than the 3.67 points found in a study targeting general hospital nurses [20] and the 3.84 points found in a study targeting clinical nurses [21]. Considering the subdomains, in this study, the nurses scored higher in the interpersonal domain than in the self-related domain, and they scored highest in terms of civility, followed by reliability and interactional justice. These results are similar to those of a study on general hospital nurses. These areas pertain to respecting end-of-life patients and recognizing their value so that they can maintain their quality of life until their death. This is considered very important for hospice nurses, who play a role in helping patients maintain their dignity [5]. Good character is a quality that all nurses who provide nursing care to patients, as well as hospice nurses, should possess as a basic qualification, and it is a very important factor that affects the quality of nursing service received by patients [20]. In addition, NC is related to nurses’ anxiety about the death of others and affects care behaviors [22]. However, research on NC is incomplete; therefore, further studies should focus on various aspects of a clinical nurse’s character.

The mean score for terminal care performance in this study was 3.18 points (range 1–4 points), similar to the mean score of 3.17 points found in a study of long-term care hospital nurses [23]. However, in a study of intensive care unit nurses [8], the mean score of performance of terminal care was 2.60 points, which was higher than that in this study. The performance of hospice nurses’ terminal care was found to be influenced by age, educational level, religion, marital status, career of hospice nurses, whether the nurse is a hospice specialist, and job satisfaction. This result differs from that in the study of intensive care unit nurses [8], where there was no difference in the performance of terminal care according to general characteristics. In addition, considering the subdomains, among the areas of terminal care, hospice nurses focused most on psychological nursing, followed by physical and spiritual nursing. However, clinical nurses and intensive care unit nurses focused most on physical, psychological [8,12], then spiritual nursing, demonstrating that hospice nurses provided more psychological nursing than nurses in other fields. This can be attributed to the fact that the goal of a hospice unit is different from that of other units. When the level of nursing for each area was compared, the level of spiritual nursing was found to be lower than that of clinical nurses [12]. This result could be explained by the results of a qualitative study dealing with the spiritual conflict of hospice nurses, who found that hospice nurses felt vulnerable when facing the death of a patient and experienced immense spiritual conflict as they were disturbed by the state of affairs before death [3]. These spiritual conflicts likely resulted in burnout among hospice nurses. Another study found that compassion fatigue caused by hospice nurses while caring for patients and families creates psychological stress [24]. For this reason, hospice nurses emphasized the need for a program to address the burnout and distress caused by compassion fatigue [24]. However, these results may be due to the characteristics of the participants in this study. In this study, nurses’ performance in terminal care was found to be higher among nurses who were older, married, and had more than 10 years of experience in the hospice ward. However, more than 50% of the participants of this study had less than three years of experience in the hospice ward. Therefore, in future research, further studies on the relationship between the experience of the hospice ward and the level of spiritual nursing is needed.

Terminal care performance was positively correlated with ANCD and NC. Religion, job satisfaction, ANCD, and NC were strong factors that influenced the performance of terminal care. In addition, NC was found to have a moderating effect on the relationship between ANCD and terminal care performance. In other words, since ANCD and NC interact, even if ANCD is low, NC buffers and improves the performance of terminal care. A previous study explored the factors influencing the performance of terminal care among intensive care unit nurses and found that stress during end-of-life nursing [8] and the stress associated with end-of-life nursing can be caused by negative attitudes or burdens of the patient or caregiver toward hospice care, interpersonal problems, or lack of knowledge and perception of hospice care [8,24,25]. Thus, even if nurses have a positive attitude toward nursing care of the dying, it is difficult for their attitudes to lead to enhanced performance unless they establish a trusting relationship with the patient’s family. At this time, NC affects the formation of smooth interpersonal relationships and resolution of conflicts in the clinical field [20]. This is because trust, listening, and responsibility, which are necessary elements for forming good interpersonal relationships, are the main components of a nurse’s clinical nursing character [11]. Therefore, a nurse with good clinical nursing character gives trust to the patient, even if the ANCD is rather poor, and has the capacity to listen to their story and practice responsible actions. These competencies will soon lead to terminal care performance. In addition, it is difficult to provide appropriate terminal nursing if there is not enough time or accurate communication between the doctor, peer nurse, or hospice team. In this situation, nurses do their best, striving to make up for their deficiencies, treating the patient sincerely and with kindness, respect, and empathy, while cooperating with other medical personnel. It is believed that, if they are polite and can be fair toward the weak, this positive attitude toward nursing care of the dying can lead to better performance of terminal care. These capabilities make up a nurse’s character [11]. In a qualitative study on the adaptation of hospice nurses to their roles, nurses assigned to the hospice ward who faced difficulties naturally and positively accepted their perception of death, understood and empathized with the patients and their families, and practiced respectful nursing [10]. It is said that they adapted to their own roles while playing the role of coordinator in their relationship with medical personnel. Therefore, to enhance the performance of terminal care by hospice nurses, it is imperative to introduce educational programs regarding end-of-life care so that nurses can develop a positive attitude toward nursing care for the dying. Additionally, when developing such a program, it is necessary to seek ways to improve clinical nursing characteristics. 

Despite these results, this study has several limitations. First, since this study selected participants through a convenience sample, the results of this study cannot be generalized to all hospice nurses. Second, since these study data were collected during the COVID-19 period, these situations may have affected the results of this study. However, since studies on NC for hospice nurses are not diverse, the results of this study emphasize the importance of education on nurses’ characteristics and provide important fundamental data for the development of policies and programs to enhance the performance of terminal care.

## 5. Conclusions

This study attempted to provide fundamental data for strategies to improve the performance of terminal care for hospice patients by examining the moderating effect of nurses’ characteristics on the relationship between the attitudes toward nursing care of the dying and the performance of hospice nurses’ terminal care. It was found that religion, job satisfaction, attitudes toward nursing care of the dying, and nurses’ characteristics affected the performance of hospice nurses’ terminal care. In addition, it was confirmed that there is a moderating effect of nurses’ character on the relationship between attitudes toward nursing care of the dying and performance of terminal care. Based on this, we suggest the following: first, it is necessary to include elements to improve nurses’ character in the educational program to improve the performance of hospice nurses’ terminal care from a clinical point of view. Second, in terms of education, education should be provided so that nursing students can have the right knowledge and awareness of hospice nursing, and, at the same time, education should be provided to improve clinical nursing characteristics. Third, in terms of research, repeated studies that expand the scope of investigation are needed, and in-depth follow-up studies on nurses’ hospice ward careers and spiritual nursing should be conducted.

## Figures and Tables

**Table 1:** General characteristics of participants (*n* = 158).

Characteristics	Categories	M ± SD or *n* (%)	MIN–MAX
Age (yr)		39.4 ± 10.8	24.0–68.0
	≤30	48 (30.4)	
	31–40	45 (28.5)	
	41–50	38 (24.1)	
	≥50	27 (17.1)	
Educational level	3-year nursing school	20 (12.7)	
	Bachelor’s degree	119 (75.3)	
	Master’s degree or higher	19 (12.0)	
Having religion	Yes	108 (68.4)	
	No	50 (31.6)	
Marital status	Yes	76 (48.1)	
	No	82 (51.9)	
Living alone	Yes	48 (22.2)	
	No	123 (77.8)	
Career of hospice nurse (yr)		3.98 ± 4.16	0–24.0
	≤1	53 (33.5)	
	2–3	42 (26.6)	
	4–10	41 (26.0)	
	≥11	22 (13.9)	
Hospice specialist nurse	Yes	38 (24.1)	
	No	120 (75.9)	
Job satisfaction	dissatisfaction	7 (4.4)	
	moderate	46 (29.1)	
	satisfaction	105 (66.5)	
Attitudes toward nursing care of the dying		3.22 ± 0.31	2.40–3.80
Performance of terminal care		3.18 ± 0.33	2.57–4.00
Nurse’s character		4.06 ± 0.40	2.87–4.97

**Table 2:** Differences in performance of terminal care according to general characteristics (*N* = 158).

Characteristics	Categories	Performance of Terminal Care	
		M ± SD	t or F (*p*)
Age (yr)	≤30 ^a^	3.06 ± 0.31	7.04 (<0.001) a < b < c,d
	31–40 ^b^	3.13 ± 0.31	
	41–50 ^c^	3.27 ± 0.30	
	≥51 ^d^	3.36 ± 0.33	
Educational level	Diploma	3.20 ± 0.30	0.36 (0.697)
	Bachelor’s degree	3.19 ± 0.34	
	Master’s degree	3.12 ± 0.29	
Having religion	Yes	3.25 ± 0.33	4.26 (<0.001)
	No	3.02 ± 0.27	
Marital status	Yes	3.28 ± 0.33	3.79 (<0.001)
	No	3.09 ± 0.30	
Living alone	Yes	3.20 ± 0.33	0.81 (0.439)
	No	3.10 ± 0.32	
Career of hospice nurse (yr)	≤1 ^a^	3.14 ± 0.39	4.95 (0.003) b,c < d
	2–3 ^b^	3.07 ± 0.26	
	4–10 ^c^	3.07 ± 0.34	
	≥11 ^d^	3.27 ± 0.32	
Hospice specialist nurse	Yes	3.28 ± 0.32	2.06 (0.054)
	No	3.28 ± 0.33	
Job satisfaction	Dissatisfaction ^a^	2.91 ± 0.37	5.09 (0.007) a < c
	Moderate ^b^	3.10 ± 0.28	
	Satisfaction ^c^	3.23 ± 0.33	

The differences and order among groups in the post-hoc comparison are indicated by superscript letters (a, b, c, d).

**Table 3:** Moderating effect of NC on the relationship between ANCD and performance of terminal care (*n* = 158).

Categories	1			2			3			4		
	*β*	t	*p*	*β*	t	*p*	*β*	t	*p*	*β*	t	*p*
Age (yr)	0.17	10.33	0.185	0.14	1.19	0.237	0.08	0.68	0.495	0.06	0.53	0.599
Having religion (ref: no)	0.17	10.92	0.057	0.13	1.54	0.127	0.14	10.80	0.074	0.15	20.10	0.037
Marital status (ref: no)	0.09	0.92	0.360	0.04	0.45	0.656	−0.04	−0.50	0.620	−0.03	−0.37	0.713
Career of hospice nurse (yr)	0.00	0.05	0.959	0.05	0.44	0.662	0.01	0.11	0.914	0.02	0.16	0.876
Hospice specialist nurse (ref: no)	0.07	0.93	0.353	0.03	0.39	0.694	0.05	0.80	0.427	0.05	0.76	0.451
Job satisfaction (ref: dissatisfaction)												
moderate	0.12	0.65	0.517	0.04	0.23	0.823	−0.08	−0.49	0.623	−0.05	−0.34	0.734
satisfaction	0.23	10.21	0.229	0.03	0.16	0.871	0.12	−0.69	0.494	−0.08	−0.47	0.640
ANCD				0.35	4.43	<0.001	0.13	10.56	0.122	0.18	20.17	0.032
NC							0.49	50.60	<0.001	0.51	60.06	<0.001
ANCD × NC										0.22	3.83	0.001
R^2^ (ΔR^2^)	0.148			0.243 (0.095)			0.372 (0.129)			0.413 (0.041)		
F	4.88			7.25			11.25			11.99		
*p*	(<0.001)			(<0.001)			(<0.001)			(<0.001)		

ANCD: Attitudes toward Nursing Care of the Dying; NC: Nurse’s Character.

## Data Availability

The data presented in this study are available upon request from the corresponding author. The data are not publicly available because of privacy concerns.
